# Evaluation of Accidental and Intentional Pediatric Poisonings: Retrospective Analysis of Emergency Medical Service Interventions in Wroclaw, Poland

**DOI:** 10.3390/nursrep14030186

**Published:** 2024-09-20

**Authors:** Jakub Wojciechowki, Michał Czapla, Marek Konop, Raúl Juárez-Vela, Joanna Rosińczuk

**Affiliations:** 1 Department Division of Scientific Research and Innovation in Emergency Medical Service, Department of Emergency Medical Service, Faculty of Nursing and Midwifery, Wroclaw Medical University, 51-616 Wroclaw, Poland; j.wojciechowski@umw.edu.pl; 2 Group of Research in Care (GRUPAC), Faculty of Health Sciences, University of La Rioja, 26006 Logrono, Spain; raul.juarez@unirioja.es; 3 Institute of Heart Diseases, University Hospital, 50-556 Wroclaw, Poland; 4 Department of Experimental Physiology and Pathophysiology, Laboratory of Centre for Preclinical Research, Medical University of Warsaw, 02-106 Warsaw, Poland; marek.konop@umw.edu.pl; 5 Department of Nursing, Faculty of Nursing and Midwifery, Wroclaw Medical University, 51-618 Wroclaw, Poland; joanna.rosinczuk@umw.edu.pl

**Keywords:** emergency medical teams, intoxication, pediatric, drug overdose, poisoning

## Abstract

**Background/Objectives:** Poisonings among children are a major cause of morbidity and mortality worldwide and present a significant challenge for emergency medical services (EMS). The aim of this study was to analyze the types of substances causing poisonings and the intention of poisonings in children, providing detailed information on the most common causes of poisonings in different age groups. **Methods**: A retrospective study was conducted analyzing medical records of pediatric patients under the care of emergency medical services (EMS) in Wroclaw, Poland, between 2015 and 2017. The study included all patients under 18 years old diagnosed with poisoning. Data collected included age, sex, cause of poisoning, location of the incident, neurological status (GCS), and the type of healthcare facility. **Results**: The study included 484 patients, with a mean age of 13 years. The largest age group was 16–18 years (44%), and the majority were female (58%). The most common causes of poisonings were alcohol (29.3%), medications (26%), and intoxicants (24.8%). Over half of the incidents occurred at home (52.5%). Intentional poisonings constituted 75% of cases, particularly involving alcohol (38.6%), intoxicants (32%), and medications (26.7%). Accidental poisonings were mainly due to household chemicals (28.7%) and carbon monoxide (27%). The differences in causes and locations of poisonings were statistically significant (*p* < 0.001). **Conclusions**: Our study showed that the most common causes of poisonings among children were alcohol, medications, and intoxicants. Poisonings most frequently occurred at home, especially with medications, while outside the home, alcohol and intoxicants were predominant. This study was not registered.

## 1. Introduction

Poisonings among children are a major cause of morbidity and mortality worldwide, although many of them are preventable [1]. Most poisonings in children are unintentional and occur at home, although there are also cases of intentional poisonings, including suicide attempts [2].

Currently, it is estimated that there are several million chemical compounds with potential toxic effects on the human body. These include widely available animal- and plant-based products, as well as various chemical substances and drugs/compounds, including medications and alcohol [3]. Medications account for more than half of potential toxic exposures in children. Poisoning can also occur as a result of ingesting or inhaling chemicals found in everyday household detergents, as well as consuming alcohol or psychoactive substances [4,5,6,7].

The epidemiology of acute poisonings in children and adolescents varies depending on geographical location, economic situation, and ethnic background [8,9,10]. For example, in developed countries with high incomes, the most common cause of acute poisonings is the misuse of available medicinal products [11,12]. In developing countries, acute poisonings are most often caused by insecticides [13]. The most common causes of poisonings in children globally include medications and vitamins, household cleaning products and chemicals, carbon monoxide, and the ingestion of poisonous plants and mushrooms [14,15,16,17]. In Poland, common causes of pediatric poisonings reported by emergency medical services (EMS) include medications, household chemicals, and alcohol [18]. This highlights the importance of safe storage practices and public education to prevent accidental poisonings.

EMS face significant challenges in responding to pediatric poisonings [19]. The unique needs of children, especially in serious or life-threatening situations, require an EMS system equipped with appropriate resources, trained personnel, and pediatric-specific protocols [20]. Effective pre- and in-hospital care and timely medical intervention are crucial to mitigating the impact of poisonings and improving outcomes for affected children [21,22].

The aim of this study was to analyze the types of substances causing poisonings and the intentionality of poisonings in children up to 18 years of age in cases where emergency medical services (EMS) were dispatched. This study aimed to provide detailed information on the most common causes of poisonings in different age groups, as well as to understand the circumstances and locations where poisonings most frequently occur.

## 2. Materials and Methods

### 2.1. Study Design and Setting

A retrospective study was conducted to analyze medical records of pediatric patients (up to 18 years of age) under the care of the emergency medical services in Wroclaw (Poland) between January 2015 and December 2017. This study focused on cases of poisonings by various substances, both accidental and intentional. The analysis included all cases where emergency medical services (EMS) were dispatched to intervene, regardless of the outcome upon arrival. The guidelines outlined by STROBE (Strengthening the Reporting of Observational Studies in Epidemiology) were followed to ensure the quality and transparency of the study [23].

### 2.2. Study Population

This study included all patients under 18 years old who were diagnosed with poisoning. A total of 484 patients met the inclusion criteria. Data collected for analysis included patients’ age, sex, cause of poisoning, location of the incident, neurological status as assessed by the Glasgow Coma Scale (GCS), and the type of healthcare facility to which the patients were transported.

### 2.3. Data Collection

The data were sourced from the Command Support System of the National Emergency Medical Service and provided by the emergency medical service in Wroclaw. The variables analyzed included both qualitative data (such as sex, cause of poisoning, location of the incident, and intention of the poisoning) and quantitative data (such as age and GCS score).

### 2.4. Statistical Analysis

Statistical analysis was performed using the Statistica 13 software (TIBCO Inc., Palo Alto, CA, USA). Descriptive statistics, including arithmetic means, medians, standard deviations, and ranges, were calculated for continuous variables. Frequencies and percentages were computed for categorical variables. The Shapiro–Wilk test was used to assess the normality of distribution for continuous variables. Chi-square (χ^2^) tests were employed to compare categorical variables, and one-way analysis of variance (ANOVA) was used for continuous variables. A significance level of α = 0.05 was set for all statistical tests.

## 3. Results

### 3.1. Characteristics of the Study Group

In the analyzed material, there were 484 individuals (n = 484), including 279 females, representing 58% of the entire group, and 205 males (42%). The ages in the entire cohort ranged from 1 month to 17 years ± 5.3 years. The largest age group was 16–18 years (n = 213; 44%), while the smallest age group was 7–12 years (n = 27; 6%). Table 1 provides the characteristics of the study group, including the distribution based on the year of intervention. The largest number of cases occurred in 2015 (n = 180; 37%). Table 1 also includes the Glasgow Coma Scale (GCS) scores, which ranged from 3 to 15 points (±5.3 points). Characteristics of the study group are presented in Table 1.

### 3.2. Causes of Poisonings

In the examined group, the most common causes of poisonings were alcohol (29.3%), medications (26%), and intoxicants (24.8%). Over half of the incidents occurred at the patients’ homes (52.5%). The majority of patients were transported to hospitals with pediatric wards (52.5%), while 24% were taken to hospitals with toxicology departments. In 18.4% of cases, patients were left at the scene after initial assessment, and the remaining 5.2% were transported by Emergency Medical Teams (EMTs) to hospitals without toxicology or pediatric wards. The causes of poisonings, along with the distribution by location, intention, type of EMT, and type of destination hospital, are presented in Table 2.

### 3.3. Types of Substances Causing Poisonings in the Study Group

The largest group of individuals were poisoned by substances of unknown origin or those not listed (43.2%). However, as many as 33.3% of the cases involved poisoning with designer drugs (synthetic psychoactive substances created to mimic the effects of controlled drugs while bypassing legal restrictions). Among the medications causing poisonings in children and adolescents, antidepressants and sedatives were the most prevalent (32.7%). A significant portion of the group was poisoned by other medications (30%) or non-steroidal anti-inflammatory drugs (NSAIDs) (25.5%). It is important to emphasize that while antidepressants and sedatives are usually prescription medications, NSAIDs are commonly available over-the-counter (OTC) drugs. The types of substances causing poisonings in the study group are presented in Table 3.

### 3.4. Characteristics of Groups by Cause of Poisoning

In the age groups of 13–15 years and 16–18 years, the most common cause of poisoning was alcohol, accounting for 34.4% and 39.4%, respectively. Additionally, there was a considerable prevalence of poisonings with intoxicants (13–15 years: 27.4%; 16–18 years: 34.7%) and medications (13–15 years: 29.9%; 16–18 years: 18.8%). In the youngest age group examined (0–6 years), the most frequent sources of poisoning were household chemicals (36.8%) and medications (31.0%). Among children aged 7–12 years, medications were the leading cause of poisoning (44.4%). Among female children, the most common causes were alcohol and intoxicants (approximately 33% each), whereas in male children, the most common causes were medications (33.3%) and alcohol (26.5%). This study revealed differences between the causes of poisonings and the time of their occurrence (Table 4).

### 3.5. Comparison of Results Regarding Intention, Location of Incident, and Recording of Incident by Causes of Poisoning

This analysis also examined the causes of poisonings in minors and their dependence on variables such as intention, location of the incident, and the type of emergency team providing assistance. The majority of poisonings occurred at home, with medications being the primary cause (37.4%). Outside the home, alcohol and intoxicants represent the leading causes of poisonings (48.3% and 29.1%, respectively). Significant differences were observed between the causes of poisonings and the location of the incident, its intention, and the place of hospitalization (*p* < 0.001).

Differences were noted between the type of poisonous substance and the intention of ingestion (*p* < 0.001). Intentional poisonings were reported primarily with alcohol (38.6%), intoxicants (32.0%), and medications (26.7%). Accidental poisonings most frequently involved household chemicals (28.7%), carbon monoxide (27.0%), and medications (24.3%).

Most patients with symptoms of poisoning from intoxicants (42.2%) and alcohol (30.2%) were admitted to hospitals with toxicology wards. Patients poisoned with medications (37.4%) were primarily admitted to hospitals with pediatric wards, while patients with symptoms of poisoning from intoxicants (40.0%) were most often taken to other hospitals (Table 5). Decisions to leave patients at the scene or hand them over to the police were made in 56.2% of alcohol poisonings.

This analysis also revealed that patients from the study group were most frequently admitted to hospitals with pediatric wards (52.5%), followed by hospitals with toxicology wards (24%). The remainder of the patients were either taken to other medical facilities or left at the scene or under police care.

## 4. Discussion

Our study on pediatric poisonings in Wroclaw from 2015 to 2017 revealed that the most common causes of poisonings were alcohol (29.3%), medications (26%), and intoxicants (24.8%). The majority of poisoning incidents occurred at home (52.5%), with 75% being intentional, particularly involving alcohol, intoxicants, and medications. These results are generally consistent with previous studies conducted worldwide. Studies from various countries show distinct patterns of pediatric poisonings that are strongly linked to age groups. Similar patterns emerge globally. In Turkey, accidental poisonings are most prevalent among younger children, while adolescents are more likely to experience intentional poisonings [9]. Likewise, in the United States, data from the American Association of Poison Control Centers (AAPCC) indicate that children under 5 years old represented 40.7% of all poison exposures, with cosmetics, household cleaning products, and personal care items being the most common substances [24]. In our study, we observed similar patterns, particularly in children aged 2–5 years, who were most frequently exposed to non-pharmaceutical substances. Furthermore, in China, preschool-aged children (45.6%) were predominantly affected by accidental poisonings, aligning with our findings where younger children were more likely to experience unintentional poisonings.

A retrospective analysis of medical records from 2006–2015 in China found that 45.6% of poisoning cases involved preschool children [25]. Similar results were obtained in Japan, where the number of poisonings in children under 6 years old was 31.5 thousand. These findings from Japan and China further underscore the global vulnerability of younger children to poisoning incidents. Our study in Wroclaw reflects a similar pattern, where children aged 2–5 years represented the group most frequently exposed to toxic substances, primarily due to accidental ingestion [26]. By 2022, in the United States, the National Poison Data System (NPDS) reported over 867,795 poison exposure cases involving children under 6 years old, with household cleaning substances (10.29%), analgesics (9.54%), and cosmetics/personal care products (9.49%) being the most frequently reported substances. In our study, we found that children aged 2–5 years were most often exposed to non-pharmaceutical agents, including household substances and intoxicants, mirroring the patterns observed in the USA [27].

Data on pediatric poisonings are also available from high-income countries. Despite the abundance of statistics, comparing them can be problematic due to different reporting criteria and geographical conditions. For example, poisoning rates in children in Australia have been consistently higher in rural areas for several years. This was due to the fact that many children with poisoning symptoms in rural areas were referred to hospitals by doctors, while in cities, the referral rate was lower (doctors managed these cases independently) [28]. One of the best indicators used for analyzing morbidity and poisoning incidence is hospital discharge data based on confirmed diagnoses. In California (USA), a two-year study based on discharge and death records found that children aged 15–17 months had the highest overall injury rate. Poisoning was the second most common cause of morbidity. Among these children, over 67% of poisoning cases were related to the ingestion of medications [29]. In Australia, children aged 0–4 years had the highest rates of poisoning, with medicinal poisonings peaking in 3-year-olds and non-medicinal poisonings most common in 2-year-olds [28]. A similar trend was observed in the USA, where children under 6 years old accounted for over 40% of all poisoning cases, with household cleaning products (10.29%), analgesics (9.54%), and cosmetics/personal care products (9.49%) being the most common substances involved [24]. In our study, we observed a comparable age-specific pattern, where children aged 2–5 years were most affected, primarily by non-pharmaceutical substances such as alcohol and intoxicants. This further highlights the similarity between global trends and local findings, with age playing a key role in the type and frequency of poisoning incidents. Lee et al., in a retrospective analysis from 2009–2019, found that in the USA, although there was a significant decrease in overall poison exposure in children, there was no observed decrease in the number of deaths due to poisoning [30].

In studies performed by Pawer et al., poisonings were most common among teenagers aged 15–19 years. They also noted an increasing percentage of hospitalizations resulting from poisonings compared to other age groups of children. In children under 1 year old, the number of poisonings is relatively small in low- and middle-income countries [31]. For example, in Nigeria, based on an analysis of medical records, out of 15,196 accidental poisoning reports in children, only 113 cases required urgent hospitalization [32]. The frequency and scope of poisonings depend on the area of the world, the socio-economic status of parents, and local activities (mainly agricultural and industrial). In a study conducted by Sahin et al., of 288 patients hospitalized in the emergency department, 51.3% were girls and 48.3% were boys. The authors found that 73% of all poisonings were due to accidental contact with a toxic substance (medications, toxic compounds, and carbon monoxide). The most common cause of poisoning was drug use, affecting 48.3% of the children studied. The second most common cause was caustic substances, affecting 23.1% of patients. In 12.5% of cases, carbon monoxide poisoning occurred. Among the drugs that most frequently caused poisoning in children were antidepressants, accounting for 11.7%. In most cases, hospitalization did not last longer than 48 h [33]. In studies conducted in Canada based on an analysis of hospitalizations due to poisonings from March 2020 to April 2020, it was found that the main substances causing poisonings were non-opioid analgesics, antipyretics, and pain relievers [31].

In a retrospective study conducted at St Mary Hospital, Corlade-Andrei et al. (2023) found that 50.19% of poisonings were voluntary, and 47.43% were accidental. The most common route of exposure was ingestion (87.1%), and poisonings primarily occurred at home (70.4%). The highest incidence was in children aged 12–18 and 1–3 years [34]. Martínez-Sánchez et al. identified significant variations in the substances causing poisonings, with carbon monoxide, medications, and household cleaning products being the most common. Their study, focusing on children in different age groups, revealed that intentional poisonings were more prevalent among adolescents, while accidental poisonings were more frequent in younger children. This aligns with our findings, where children aged 2–5 years were predominantly affected by accidental poisonings involving non-pharmaceutical substances. These international comparisons underscore the importance of preventive measures and increased parental supervision, particularly in younger age groups, to mitigate the risk of accidental poisonings [35].

Considering that 75% of poisonings were intentional, there is a significant risk associated with storing psychoactive substances at home. It can be assumed that the home is the main source of access to these substances for the studied individuals. Similar conclusions were reached by other researchers, emphasizing that proper securing of hazardous substances at home contributes to reducing the number of poisonings and significantly mitigating their effects [36]. Proper securing of medications and other substances can reduce the incidence of poisonings in children by up to 20% [37]. Azab et al., during a five-year study of 38,470 pediatric poisoning cases in Egypt, found that 52% of cases involved children under 6 years old, with non-pharmaceutical substances, such as corrosives and pesticides, being the most common agents. This mirrors the findings from our study, where children aged 2–5 years were similarly exposed to non-pharmaceutical substances, further supporting the global trend of unintentional poisonings in younger children [38]. Based on the above data, it can be concluded that poisonings in preschool-aged children are mainly accidental and result from the ingestion of non-pharmaceutical substances. In contrast, poisonings in the adolescent group were mostly intentional. Our study spans the years 2015–2017, and during this period, shifts in public health policies and the availability of substances likely influenced trends in pediatric poisonings. Li et al. noted that despite a decrease in overall poisoning exposure rates in children, fatalities due to pharmaceuticals, particularly among adolescents, remained stable. This was often linked to intentional poisonings associated with suicidal behavior, highlighting a growing concern over the accessibility of medications [30]. Similarly, Mottla et al. emphasize the role of preventive strategies such as education on safe storage of medications and improved regulatory frameworks to limit access to harmful substances. While such interventions have proven effective in high-income countries, the study suggests that changes in healthcare accessibility during the study period might have impacted poisoning trends in various regions [16]. Furthermore, Johnston et al. observed fluctuations in substance use among adolescents that corresponded with changes in public perception and policy. For example, stricter regulations on alcohol sales and increased awareness of the dangers of certain intoxicants led to changes in their availability, potentially influencing the patterns of intentional poisonings observed in our study [39].

### 4.1. Study Limitation

This study had several limitations that may affect the generalizability of the findings. Firstly, socio-economic factors such as parental education, socio-economic status, and cultural attitudes toward substance use were not included in the analysis. These variables may play a significant role in determining the likelihood of pediatric poisonings, and their exclusion may limit the ability to fully understand the underlying causes and risk factors. Future studies should consider including these socio-economic variables to provide a more comprehensive understanding of pediatric poisonings.

Secondly, although we acknowledge that public health policies and changes in substance availability or social behaviors may have influenced poisoning patterns, these factors were not systematically tracked during the study period. Shifts in public health interventions or the accessibility of substances from 2015 to 2017 may have impacted the incidence and types of poisonings. The absence of specific epidemiological data on these changes may confound the interpretation of the results. This is an important consideration, as trends in substance use and access to healthcare may have evolved since the study period. Future research should monitor public health policies and social trends to better contextualize findings.

Additionally, there is a potential for “immortal bias” in our study, as we only included cases of children who survived until EMS intervention. This limitation may exclude cases where a child did not survive long enough to receive EMS assistance, thus potentially skewing the data toward less severe poisonings. We acknowledge that this could affect the overall validity of the results, particularly in relation to more severe poisoning cases.

Finally, our analysis was limited to data available from the Wroclaw emergency medical service, which may not fully represent the situation in other regions or countries. While we believe the data provide valuable insights to medical personnel, the lack of broader national or international data further limits the generalizability of the findings. Expanding future research to include data from other regions would allow for a more complete understanding of pediatric poisonings.

### 4.2. Practical Implications

The findings of this study have important implications for clinical nursing practice, particularly in pediatric and emergency care settings. Nurses are at the forefront of patient care in cases of pediatric poisonings and play a critical role in both prevention and intervention. One of the key takeaways from this research is the high prevalence of accidental poisonings among young children, especially within the home environment. This emphasizes the importance of nurses in educating parents and caregivers about safe storage practices for medications, alcohol, and household chemicals. Given that over half of the poisonings occurred at home, preventive education spearheaded by nurses can significantly reduce the risk of accidental exposure to toxic substances. Through outreach programs and in clinical settings, nurses can guide families on the necessity of childproofing their homes and properly securing hazardous materials, thereby directly influencing the reduction in such incidents.

In addition to their preventive role, nurses are essential in the early identification and management of intentional poisonings, particularly in adolescents. This study highlights the substantial number of intentional poisonings, suggesting the need for routine mental health screenings as part of nursing practice, especially in pediatric and school healthcare settings. By integrating mental health assessments into their care routines, nurses can identify at-risk individuals early, providing necessary interventions or referrals to mental health professionals. The close relationship nurses often develop with their patients enables them to detect early signs of distress or risky behaviors, which may lead to intentional poisonings. Addressing these underlying issues can not only prevent future poisonings but also improve the overall wellbeing of the child or adolescent.

Furthermore, in the emergency care context, nurses are often the first healthcare providers to respond to poisoning cases. This research provides valuable insights into the most common substances involved in pediatric poisonings, such as alcohol, medications, and intoxicants, which can enhance nurses’ ability to respond quickly and effectively. Having a clear understanding of the substances most frequently responsible for poisonings allows nurses to make informed decisions about treatment options, ensuring that patients receive timely and appropriate care. This study also underscores the need for refined clinical protocols specific to pediatric poisonings, which can guide nurses in managing these cases more efficiently and safely.

Lastly, the implications of this research extend to nursing advocacy for broader public health measures. Nurses, drawing on their clinical experience and the data presented in this study, are well positioned to advocate for policy changes that enhance child safety. These may include stronger regulations on the sale of alcohol and intoxicants to minors, as well as mandatory child-resistant packaging for medications and household chemicals. By championing these causes, nurses contribute to a safer environment for children, reducing the incidence of both accidental and intentional poisonings.

## 5. Conclusions

Our study showed that the most common causes of poisonings among children were alcohol, medications, and intoxicants, with alcohol poisoning being predominant among adolescents over the age of 13. In the youngest children (under 6 years old), the most common causes of poisonings were household chemicals and medications. A significant majority of poisonings were intentional, particularly in cases involving alcohol, intoxicants, and medications. Poisonings most frequently occurred at home, especially with medications, while outside the home, alcohol and intoxicants were the leading causes. Patients were most often admitted to hospitals with pediatric wards, while those admitted to hospitals with toxicology wards were primarily poisoned by intoxicants and alcohol.

## Figures and Tables

**Table 1:** Characteristics of the study group.

	Study Group n = 484	
Age [years]	Mean	13
	Me	15
	Min–Max	0.0–17.0
	SD	5.3
Age [categories]	0–6 lat	n = 87; 18%;
	7–12 lat	n = 27; 6%;
	13–15 lat	n = 157; 32%;
	16–18 lat	n = 213; 44%;
GCS [points]	Mean	14.1
	Me	15
	Min–Max	3.0–15.0
	SD	2.1
Sex	female	n = 279; 58%;
	male	n = 205; 42%;

n—number of patients, %—percentage, Me—median, min—minimum value, max—maximum value, SD—standard deviation.

**Table 2:** The causes of poisonings, along with the distribution by location, intention, type of EMT, and type of destination hospital.

Variables		Study Group	
		n	%
Cause of poisoning	Alcohol	142	29.3
	Medications	126	26
	Intoxicants	120	24.8
	Household chemicals	33	6.8
	Plant toxins	32	6.6
	Carbon monoxide	31	6.4
Location of incident	Home	254	52.5
	Outside home	230	47.5
Intention of Poisoning	Intentional	363	75
	Accidental	115	23.8
Destination Hospital	Hospital with pediatric department	254	52.5
	Hospital with toxicology department	116	24
	Left at the scene, no consent for transport or handed over to police	89	18.4
	Other hospitals, emergency room/emergency department	25	5.2

n—number of patients; %—percent.

**Table 3:** Types of substances causing poisonings in the study group.

Variables		Study Group	
		n	%
Intoxicants	Other or unknown	35	43.2
	Designer drugs	27	33.3
	THC	9	11.1
	Opioids	6	7.4
	Amphetamines	4	4.9
Medications	Antidepressants and sedatives	36	32.7
	Other or unknown	33	30
	NSAIDs	28	25.5
	Dextromethorphan	10	9.1
	Benzodiazepines	3	2.7

%—precent; n—number of patients; %—percentage, THC—tetrahydrocannabinol, NSAIDs—non-steroidal anti-inflammatory drugs.

**Table 4:** Comparison of results regarding group characteristics by causes of poisoning.

Variables			Cause of Poisoning						*p*-Value *
			Carbon Monoxide	Alcohol	Intoxicants	Medications	Household Chemicals	Plant Toxins	
Age [years]	0–6	n	18	1	0	27	32	9	<0.001
		%	20.7	1.1	0	31	36.8	10.3	
	7–12	n	6	3	3	12	1	2	
		%	22.2	11.1	11.1	44.4	3.7	7.4	
	13–15	n	4	54	43	47	0	9	
		%	2.5	34.4	27.4	29.9	0	5.7	
	16–18	n	3	84	74	40	0	12	
		%	1.4	39.4	34.7	18.8	0	5.6	
Sex	female	n	8	68	67	33	18	11	<0.001
		%	3.9	33.2	32.7	16.1	8.8	5.4	
	male	n	23	74	53	93	15	21	
		%	8.2	26.5	19	33.3	5.4	7.5	

* χ^2^ test; n—number of patients; %—percentage of patients.

**Table 5:** Comparison of results regarding intention, location of incident, and recording of incident by causes of poisoning.

Variables			Cause of Poisining						*p*-Value *
			Carbon Monoxide	Alcohol	Intoxicants	Medications	Household Chemicals	Plant Toxins	
Location of incident	Home	n	30	31	53	95	30	15	<0.001
		%	11.8	12.2	20.9	37.4	11.8	5.9	
	Outside home	n	1	111	67	31	3	17	
		%	0.4	48.3	29.1	13.5	1.3	7.4	
Intention of Poisoning	Intentional	n	0	140	116	97	0	10	<0.001
		%	0	38.6	32	26.7	0	2.8	
	Accidental	n	31	1	2	28	33	20	
		%	27	0.9	1.7	24.3	28.7	17.4	
	No information	n	0	1	2	1	0	2	
		%	0	16.7	33.3	16.7	0	33.3	
Destination Hospital	Hospital with toxicology department	n	2	35	49	25	1	4	0.001
		%	1.7	30.2	42.2	21.6	0.9	3.4	
	Hospital with pediatric department	n	27	52	47	95	24	9	
		%	10.6	20.5	18.5	37.4	9.4	3.5	
	Other hospitals with emergency room/emergency department	n	2	5	10	2	1	5	
		%	8	20	40	8	4	20	
	Left at the scene, no consent for transport, or handed over to police	n	0	50	14	4	7	14	
		%	0	56.2	15.7	4.5	7.9	15.7	

* χ^2^ test; n—number of patients; %—percentage of patient.

## Data Availability

The data that support the findings of this study are available from the corresponding author upon reasonable request.

## Public Involvement Statement

No public involvement in any aspect of this research.

## Guidelines and Standards Statement

This manuscript was drafted against the Strengthening the Reporting of Observational studies (STROBE).
